# ﻿The first record of the genus *Dichodontocis* Kawanabe, 1994 (Coleoptera, Ciidae) from China, with the description of a new species and its larva

**DOI:** 10.3897/zookeys.1218.130088

**Published:** 2024-11-20

**Authors:** Nan Li, Ji-Shan Xu

**Affiliations:** 1 College of Agriculture and Biological Science, Dali University, Dali, Yunnan 671003, China Dali University Dali China; 2 Co-Innovation Center for Cangshan Mountain and Erhai Lake Integrated Protection and Green Development of Yunnan Province, Dali University, Dali, Yunnan 671003, China Dali University Dali China

**Keywords:** Minute tree-fungus beetle, Ciini, morphology, taxonomy, Guangdong

## Abstract

The genus *Dichodontocis* Kawanabe, 1994 is newly recorded from China, and a new species, *Dichodontocisguangzhouensis***sp. nov.** and its larva, is described and illustrated from Guangdong Province. We provide habitat and host fungi photos of the new species and a key to all described species of the genus.

## ﻿Introduction

The genus *Dichodontocis* was described by Kawanabe (1994) based on a new species named *Dichodontocisuncinatus* (Kawanabe, 1994) from southwestern Japan, which has unique characteristics of the 10-segmented antennae, the bidentate apex and the serrated outer margin of protibiae, and the laminate prosternal process (Kawanabe 1994). It belongs to the tribe Ciini of the subfamily Ciinae because of the globulous procoxae and the presence of the metaventrital medio-longitudinal groove (Lawrence 1971; Kawanabe 1994). Then the genus *Dichodontocis* was redescribed and a new species named *D.queenslandicus* (Lawrence, 2016) was described by Lawrence in 2016. The genus *Dichodontocis* has the following main characteristics (Lawrence 2016): 1) dual vestiture consisting of bristles and fine hairs; 2) outer edge of protibia lined with fixed spines and bearing two teeth at the outer apical angle; 3) spinose meso- and metatibial apices; 4) short, weakly biconcave and subcarinate prosternum with laminate prosternal process; 5) dual, seriate elytral punctation; and 6) 10-segmented antennae.

In the present paper, a new species of the genus *Dichodontocis*, *D.guangzhouensis* sp. nov., and its larva are described and illustrated.

## ﻿Material and methods

The type specimens of the new species described herein are deposited in the Biological Science Museum, Dali University, Yunnan, China.

Adults and larvae were examined under an Olympus SZX7 stereomicroscope (Olympus, Shinjuku, Japan). The adult genitalia were dissected, and then temporarily fixed on glass slides by glycerol. Specimens were photographed and measured using the Keyence VHX-7000 free-angle observation system (Keyence, Osaka, Japan). Adult specimens were pinned, and the genitalia were preserved in glycerol. Habitus images were taken using a digital camera (Canon EOS 60D). All figures were post-corrected with Adobe Photoshop CS6 software.

The terminology of adults follows Lawrence and Lopes-Andrade (2010) and Lawrence (1974, 2016). The structure terminology of hind wings follows Lawrence et al. (2021, 2022). The structure terminology of larvae follows Lawrence (1974). The following abbreviations are adapted (Lopes-Andrade and Grebennikov 2015): TL (total length, excluding head), PL (pronotal length along midline), PW (greatest pronotal width), EL (elytral length along the midline), EW (greatest width of both elytra), GD (greatest depth of body measured in lateral view), GW (greatest diameter of compound eye), BW (basal width of scutellar shield).

## ﻿Taxonomy

### ﻿ Dichodontocis guangzhouensis sp. nov.

Taxon classificationAnimaliaColeopteraCiidae

B4A69A0A-6660-5CF5-A205-80DD5EA25E32

https://zoobank.org/163A1D96-3556-4578-A340-85D0291607EA

Figs 1 , 2 , 3

#### Type material.

***Holotype***: China • male, “China: Guangdong, Guangzhou (广州), Huangpu District (黄埔区), Tianlu Lake Forest Park (天鹿湖森林公园), 23°12'N, 113°25'E, 303 m, 05.XI.2023, leg. Da-Rui Mo”. ***Paratypes***: China • 3 males, 6 females, same data as the holotype.

#### Diagnosis.

**Figure 1.**
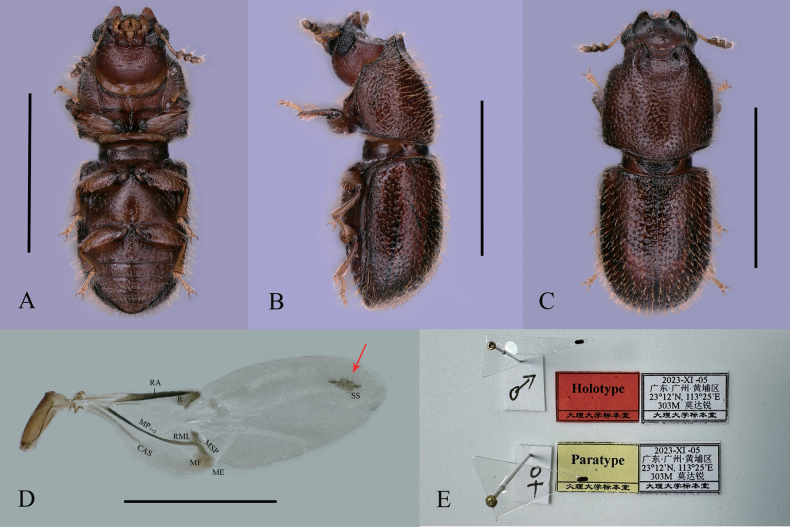
*Dichodontocisguangzhouensis* sp. nov. **A–C** holotype **D** male paratype **A–C** ventral, lateral and dorsal views, respectively **D** right hind wing of male, (dorsal view, RA: radius anterior, R: radial cell, MP_1+2_: media posterior, branches 1 and 2, RML: radiomedial loop, MSP: medial spur, CAS: cubitoanal strut, MF: medial fleck, ME: medial embayment, SS: support sclerite) **E** labels of holotype and one paratype. Scale bars: 1 mm (**A–D**).

**Figure 2.**
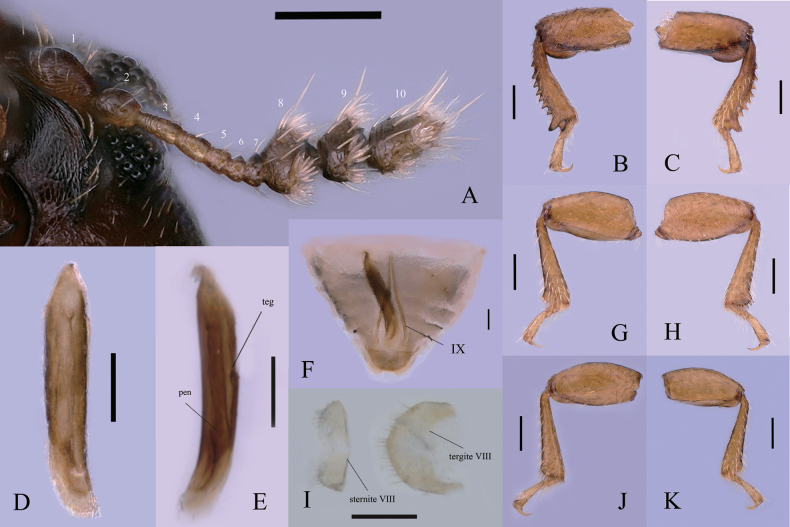
*Dichodontocisguangzhouensis* sp. nov., male paratypes **A** antenna **B** protibia, ventral view **C** protibia, dorsal view **D** tegmen and penis, dorsal view **E** tegmen (teg) and penis (pen), lateral view **F** terminal segments of the abdomen, including the aedeagus and pregenital segments, the abdominal ventrites have been removed, leaving only the eighth sternite, ventral view **G** mesotibia, ventral view **H** mesotibia, dorsal view **I** sternite VIII, ventral view, and tergite VIII, dorsal view **J** metatibia, ventral view **K** metatibia, dorsal view. Scale bars: 0.1 mm (**A–K**).

**Figure 3.**
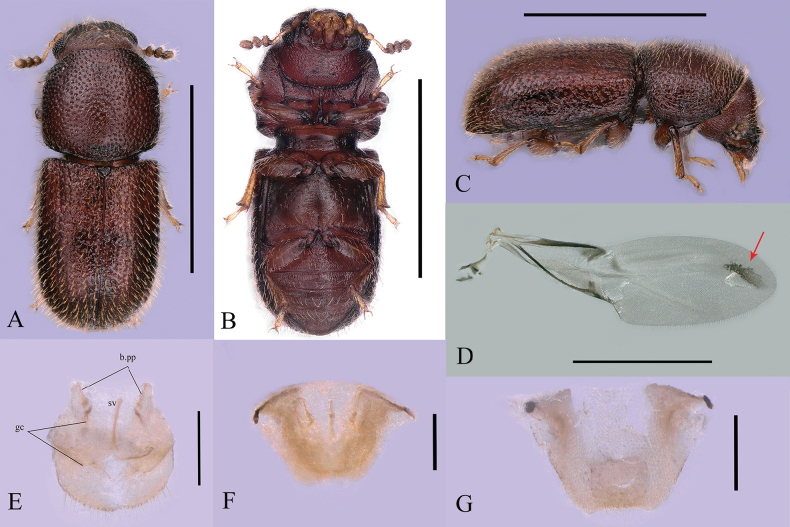
*Dichodontocisguangzhouensis* sp. nov., female paratype **A–C** dorsal, ventral, and lateral views, respectibly **D** right hind wing of female, dorsal view **E** female genitalia (ventral view, b.pp = paraproctal baculi; gc = gonocoxites; sv = spiculum ventral) **F** female genitalia, dorsal view **G** tergite VIII, dorsal view. Scale bars: 1 mm (**A–D**); 0.1 mm (**E–G**).

This new species is similar to *D.uncinatus* from southern Japan. From the illustration of the holotype (Kawanabe 1994), the elytral setae appear to be more clearly seriate and ordered in that species than in *D.guangzhouensis* sp. nov., both anal veins of hind wings are absent, but in *D.guangzhouensis* sp. nov. the hind wings have apical color spots (Figs 1D, 3D) and the impression of the anterior margin of pronotum of *D.guangzhouensis* appears broadly and deeply impressed and extends posteriorly, distinctly beyond the bases of the horn, while in *D.uncinatus*, from the illustration of the holotype (Kawanabe 1994), the impression of the anterior margin of pronotum is only between the bases of the horn and much weaker than in *D.guangzhouensis* sp. nov.. The terminalia are somewhat different. In *D.uncinatus*, the eighth abdominal sternite has the posterior margin slightly emarginate at the middle, and the anterior margin strongly emarginate. In the new species, however, the eighth abdominal sternite with the posterior margin is broadly emarginate in the middle, anterior margin is not emarginate (Fig. 2I) in the middle. The tegmen is broad at the base and gradually narrower near the end in *D.uncinatus*. In the new species, the tegmen is broad at the base, from base 1/5–4/5, the sides are nearly parallel, and gradually narrower near the end, slightly curving.

The new species is also similar to *D.queenslandicus* from northern Queensland. The size and dimensions of the two species are similar. But there are some differences between the two species. Head relatively and largely exposed, partially visible from above in *D.queenslandicus*. Head almost entirely exposed, visible from above in the new species. The impression of the anterior margin of the pronotum of *D.guangzhouensis* sp. nov. appears broadly and deeply impressed and extends posteriorly, distinctly beyond the bases of the horn, while in *D.queenslandicus*, from the illustration of the holotype (Lawrence 2016), the impression of the anterior margin of pronotum is slightly weaker and more in front than in *D.guangzhouensis* sp. nov. The tegmen of *D.queenslandicus* is 4 times as long as wide, and the pens is 4 times as long as wide. The tegmen of the new species is 6.4 times as long as wide, pens 8.0 times as long as wide. The sternite VIII of the *D.queenslandicus* is the posterior margin slightly emarginate in the middle, the eighth abdominal sternite of the new species with the posterior margin is broadly emarginate in the middle, anterior margin is not emarginate (Fig. 2I) in the middle.

#### Description of the adult.

With the characters of the genus. Male. Fully pigmented adult. Measurements in mm: TL 1.511, PL 0.678, PW 0.695, EL 0.833, EW 0.717, GD 0.636. Ratios: PL/PW 0.98, EL/EW 1.16, EL/PL 1.23, GD/EW 0.89, TL/EW 2.11.

Body (Fig. 1C) elongate, convex, reddish brown to dark brown; dorsal setae, tarsi, antennae (except club), maxillary palpus and labial palpus are pale yellow; dorsal vestiture dual, consisting of long and short erect setae.

Head wider slightly than long, partially visible from above, with sparse punctures and dual setae, frontoclypeal strongly elevated forming a pair of broad-based, reflexed at each side subtriangular plates, behind eyes. Compound eyes are finely facetted and suboval, each bearing approximately 70 ommatidia; GW 0.17 mm. Antennae (Fig. 2A) bearing 10 antennomeres with the following lengths (in mm): 0.07, 0.05, 0.04, 0.03, 0.02, 0.02, 0.02, 0.05, 0.05, 0.08. Mandibles are asymmetrical (misaligned), with well-developed, transversely ridged molae.

Pronotum (Fig. 1C) is as long as broad and parallel-sided, which is slightly raised in the middle with shallow and fine punctures bearing short and long dual setae, erect. Punctures are separated by a distance equal to one to two diameters. Lateral margins (Fig. 1B, C) are narrow, and not visible for their entire lengths from above. Anterior edge produced forward and upturned into two small lateral projections. The anterior edge deeply and broadly emarginate in the middle just like a horseshoe, and the impression of the anterior margin of the pronotum extends posteriorly, distinctly beyond the bases of the horn. Anterior angles (Fig. 1B) obtuse. Posterior margin (Fig. 1B) feebly bisinuous posterior angles rounded.

Scutellar shield developed, with a few punctures and setae; subtriangular, BW 0.13 mm.

Elytra (Fig. 1C) 1.2 times as long as broad, 1.3 times as long as pronotum; punctures rough; setae dual short and long, inclined and suberect; sides subparallel in basal 2/3, then gradually convergent apical.

Hind wings (Fig. 1D) are fully developed, nearly pellucid. The venation type is folding patterns. Strongly reduced venation patterns of the clavus, anal vein absent. A medial field with one vein and a small fleck, a medial embayment, a small support sclerite near the wing apex; with a distinct cubitoanal strut and radiomedial loop. The hind wings are 3.3 times as long as broad, the widest part is in the middle, shrinking to the sides.

Protibia (Fig. 2B, C) expanded to the apex, outer apical angle with 2 acute teeth and dentate along the outer edge which are shorter near the base and gradually becoming longer towards the apex. Meso-and metatibia (Fig. 2G, H, J, K) expanded forming a rounded lobe lined with socketed spines.

Prosternum (Fig. 1A) is weakly biconcave and subcarinate; the prosternal process is flaked and slender somewhat slightly broadened near the apex, slightly higher than the procoxae (best seen in lateral view).

Metaventrite (Fig. 1A) is convex but slightly emarginate in the middle, with sparse punctuation and bristles; discrimen nearly one-fourth length of ventrite. Abdominal ventrites (Fig. 1A) with fine setae and shallow puncture, the surface between them microreticulate; the first abdominal ventrite is 2.2 times as long as 2^nd^, the ventrites bearing a small, circular, not obvious and marginally pubescent fovea. The length of the ventrites (in mm) is as follows: 0.202, 0.093, 0.077, 0.072, 0.078.

Aedeagus 4.9 times as long as ventrite 5. Tegmen (Fig. 2D, E) is slender 6.4 times as long as the widest, widest at the base and gradually narrower near the apex with the basal end subacute like a hook; narrowed gradually from base to apex; apex slightly bent. Penis (Fig. 2E) is shorter and narrower than the tegmen, 8.0 times as long as the widest, with a subacute apex and moderately long, sides parallel from basal to four fifth, then just like the rhombus near the end. The basal piece is weakly sclerotized, much longer than a broad, like a narrow horseshoe. The sternite VIII (Fig. 2I) with the posterior margin broadly emarginate in the middle, with several short hairs on each side, anterior margin not emarginate. The tergite VIII (Fig. 2I) with the posterior margin not emarginate in the middle, anterior margin strongly emarginate.

**Female** (Fig. 3). Similar to male, except for the following points: frontoclypeal without strongly subtriangular plates; pronotum with anterior edge broadly rounded, without projections; first abdominal ventrites without a sex patch. The eighth abdominal sternite of females is different from males. The posterior margin is not emarginate in the middle which is flat and the anterior margin is strongly emarginate in the female. Female genitalia (Fig. 3E, F) is as long as wide, widest at the middle; paraprocts (Fig. 3E) is 0.85 times as long as gonocoxites.

***Measurements*.** Males (*n* = 3, including the holotype; mm): TL 1.48–1.51 (1.50 ± 0.02); PL 0.66–0.68 (0.67 ± 0.01); PW 0.70–0.71 (0.70 ± 0.01); EL 0.83–0.84 (0.87 ± 0.01); EW 0.70–0.72 (0.71 ± 0.01); GD 0.63–0.64 (0.64 ± 0.01).

Females (*n* = 6; mm): TL 1.18–1.48 (1.33 ± 0.10); PL 0.46–0.60 (0.55 ± 0.06); PW 0.51–0.65 (0.59 ± 0.06); EL 0.72–0.90 (0.78 ± 0.07); EW 0.58–0.70 (0.65 ± 0.44); GD 0.56–0.74 (0.67 ± 0.08).

#### Distribution.

Tianlu Lake Forest Park, Guangzhou, Guangdong, China.

#### Host fungi.

Unidentified Polyporaceae (Fig. 6).

#### Etymology.

The specific name *guangzhouensis* is taken from the type locality, Guangzhou.

#### Description of larva.

**Figure 4.**
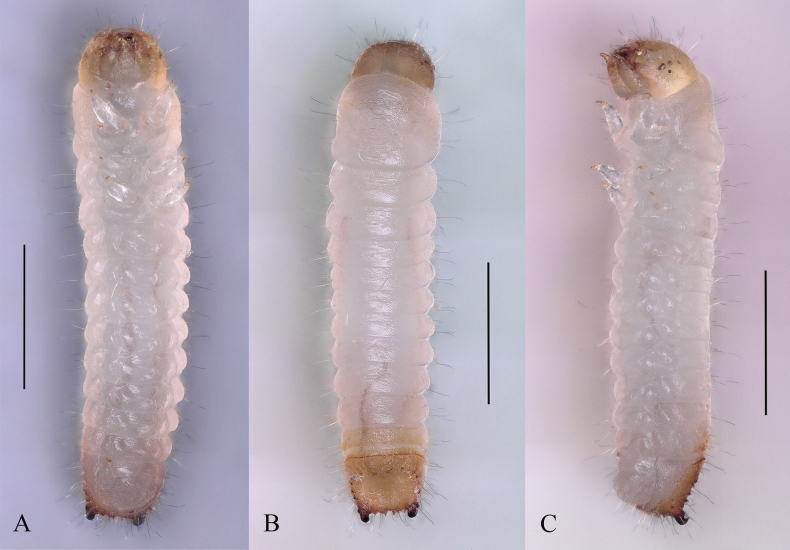
Larva of *Dichodontocisguangzhouensis* sp. nov. **A** ventral view **B** dorsal view **C** lateral view. Scale bars: 1 mm (**A–C**).

**Figure 5.**
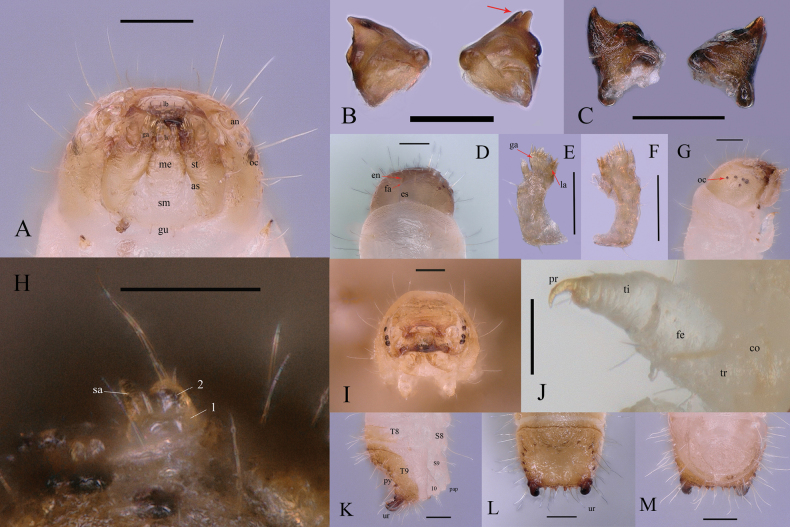
Details of the larva of *Dichodontocisguangzhouensis* sp. nov. **A** head and mouthparts (ventral view, lb: labrum, li: ligula, ga: galea, an: antenna, me: mentum, sm: submentum, gu: gula, st: stipes, as: articulating sclerite, oc: ocelli) **B** mandibles, ventral view **C** mandibles, dorsal view **D** head (dorsal view, en: endocarina, es: epicranial stem, fa: frontal arms) **E** maxillary (dorsal view, ga: galea, la: lacinia) **F** maxillary, ventral view **G** head (lateral view, oc: ocelli) **H** antenna (sa: sensory appendix) **I** head, frontal view **J** leg (co: coxa, tr: trochanter, fe: femur, ti: tibiotarsus, pr: pretarsus) **K** apex of abdomen (lateral view, T8: tergum 8, S8: sternite 8, T9: tergum 9, S9: sternite 9, ur: urogomphi, py: pygidium, pap: papillae) **L** apex of abdomen (dorsal view, ur: urogomphi) **M** apex of abdomen, ventral view. Scale bars: 0.1 mm (**A–G, I, K–M**); 0.05 mm (**H, J**).

Figs 4, 5. ***Larva material*.** “China: Guangdong, Guangzhou (广州), Huangpu District (黄埔区), Tianlu Lake Forest Park (天鹿湖森林公园), 23°12'N, 113°25'E, 303 m, 05.XI.2023, leg. Da-Rui Mo”. We are sure that the polypore basidiomes contain only one species of insect, after rearing the larvae in the laboratory, it was found that they developed into adults of the new species successfully; however, their stadiums were not accurately recorded.

**Figure 6.**
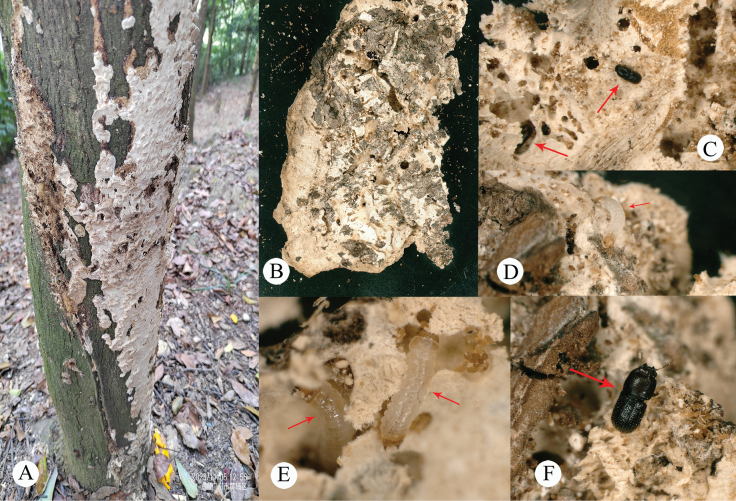
Habitat of *Dichodontocisguangzhouensis* sp. nov. **A** trunk with polypore basidiomes **B** host fungi, unidentified Polyporaceae**C–F** Live specimen containing larvae and adults of *D.guangzhouensis* sp. nov. on the surface of a polypore basidiome.

This description is based on a later instar larva (Fig. 4); 1.69 mm long, 0.35 mm broad, head-capsule of 0.31 mm wide. The body is opal and translucent except for the head, pretarsus (claw) and pygidium, which are light yellow to dark brown.

Body elongate, more or less parallel-sided, subcylindrical, slightly curved ventrally. Surfaces are relatively smooth except for the mouth frame and tips of urogomphi, rarely with light tergal plates on most segments, smooth with vestiture of scattered long and short setae.

Head subspherical, protracted and moderately to strongly declined (hypognathous); posterior edge not emarginate. The epicranial stem (Fig. 5D) is long with median endocarina (Fig. 5D) beneath it; frontal arms (Fig. 5D) are Y-shaped, with five stemmata on each side.

Antennal (Fig. 5H) insertion is a well-developed and concealed maxillary articulatory membrane. Antennae are very short with 2 segments which have a sensorium on the first segment and a long seta on the apical antennomere. The 1^st^ segment is wider and shorter than the 2^nd^.

Mandibles (Fig. 5B, C) are large, robust, asymmetrical, and bidentate (one is large and the other is small), with a simple and transversely cutting edge.

Ventral mouthparts (Fig. 5A) retracted; stipes longer than wide; maxillary articulating area (Fig. 5A) reduced; galea (Fig. 5A, E, F) rounded; lacinia (Fig. 5E) represented by a short, truncate, subapical, lobe on the dorsal surface (ventral side not visible); palp (Fig. 5A) 3-segmented.

Labium (Fig. 5A) with submentum, mentum, bearing short ligula and 2-segmented palps. Hypopharyngeal sclerome absent.

Hypostomal rods absent; ventral epicranial ridges present.

Gula (Fig. 5A) is wider than long, fused to the submentum.

Thoracic terga without transverse carinae or rows of asperities. Prothorax (Fig. 4) is only slightly larger than meso- or metathorax.

Prosternum without special armature. Thoracic legs (Fig. 5J) are short and broad, subequal; 5-segmented with pretarsus (claw), bearing a few setae; coxae relatively close together.

The length of the abdomen is more than twice as long as thorax; terga and sterna without patches.

Terga IX (Fig. 5K, L) is slightly transverse and longer and has variously modified, with concave and heavily sclerotized disc and with a pair of upturned urogomphi whose color is deepened from brown to black. There are 4–6 dark-colored, small protrusions between the urogomphi. There are also five protrusions on each side of the terga. Segment X (Fig. 5K) is transverse, posteroventrally oriented. It is only about ½ as long as terga IX; anal opening transverse. Spiracles are annular.

***Measurements*. *Later instar larva*** (*n* = 5, mm): TL 1.33–1.75 (1.61 ± 0.17); Wide 0.31–0.35 (0.34 ± 0.02).

#### Comments.

Currently, there are only three species of *Dichodontocis* known in the world. The type species, *D.uncinatus* is distributed in southwestern Japan (Yakushima island). *Dichodontocisqueenslandicus* occurs in a rainforest in Australia (Queensland). The new species is known from Guangzhou (Tianlu Lake Forest Park), China. As they are distributed in hot, rainy environments, more *Dichodontocis* species may eventually be found in tropical areas of Asia and Australia.

### ﻿Key to the described *Dichodontocis* species

Based on Kawanabe 1994 and Lawrence 2016.

**Table:** 

1	Elytral vestiture is clearly seriate, the impression of the anterior margin of pronotum is weak and only between the bases of the horn, the sternite VIII has the posterior margin slightly emarginate at the middle, and the anterior margin strongly emarginate	***D.uncinatus***
–	Elytral vestiture is slightly irregular, The impression of the anterior margin of pronotum is deeper and broader and extends posteriorly, distinctly beyond the bases of the horn	**2**
2	The posterior edge of sternite VIII is slightly narrowly emarginate in the middle	***D.queenslandicus***
–	The eighth abdominal sternite with the posterior margin broadly emarginate in the middle	***D.guangzhouensis* sp. nov.**

## Supplementary Material

XML Treatment for Dichodontocis guangzhouensis

## ﻿Additional information

### Author ORCIDs

Nan Li https://orcid.org/0009-0001-0013-6536

Ji-Shan Xu https://orcid.org/0000-0001-8903-0078

### Data availability

All of the data that support the findings of this study are available in the main text.
